# Socioeconomic inequalities in access barriers to seeking health services in four Latin American countries

**DOI:** 10.26633/RPSP.2020.11

**Published:** 2020-03-04

**Authors:** Natalia Houghton, Ernesto Bascolo, Amalia del Riego

**Affiliations:** 1 Pan American Health Organization World Health Organization WashingtonDC United States of America Pan American Health Organization/World Health Organization, Washington, DC, United States of America.

**Keywords:** Health services accessibility, socioeconomic gradient in health, health care reform, Latin America, Colombia, El Salvador, Paraguay, Peru, Accesibilidad a los servicios de salud, gradiente socioeconómico de salud, reforma de la atención de salud, América Latina, Colombia, El Salvador, Paraguay, Perú, Acesso aos serviços de saúde, gradiente socioeconômico de saúde, reforma dos serviços de saúde, América Latina, Colômbia, El Salvador, Paraguai, Peru

## Abstract

**Objective.:**

To present summary measures of socioeconomic inequalities in access barriers to health services in Colombia, El Salvador, Paraguay, and Peru.

**Methods.:**

This cross-sectional study used data from nationally - representative household surveys in Colombia, El Salvador, Peru, and Paraguay to analyze income-related inequalities in barriers to seeking health services. Households that reported having a health problem (disease/accident) and not seeking professional health care were considered to be facing access barriers. The measures of inequality were the slope index of inequality and relative index of inequality.

**Results.:**

Inequality trends were mixed across the four countries. All showed improvement, but large inequality gaps persisted between the highest and lowest income quintiles, despite health care reforms. Relative inequality gaps were highest in Colombia (60%), followed by Paraguay (30%), Peru (20%), and El Salvador (20%).

**Conclusions.:**

The effect of national policy initiatives on equity to accessing health services should be the object of future analysis. There is also a need for research on national and regional monitoring of access barriers and explanatory factors for why people do not seek care, even when having a health problem.

## RESUMEN

**Objetivo.:**

Presentar algunas mediciones de las desigualdades socioeconómicas que representan obstáculos para el acceso a los servicios de salud en Colombia, El Salvador, Paraguay y Perú.

**Métodos.:**

En este estudio transversal se tomaron datos de encuestas realizadas en hogares representativas a nivel nacional en Colombia, El Salvador, Perú y Paraguay. Mediante estos datos se analizaron las desigualdades en los ingresos como obstáculos para recurrir a los servicios de salud. Los hogares donde se informó algún problema de salud (enfermedad o accidente) y sus habitantes no recurrieron a ningún tipo de atención profesional fueron considerados como población que enfrenta obstáculos para acceder a estos servicios. Para medir la desigualdad se usó el índice de desigualdad de la pendiente y el índice relativo de desigualdad.

**Resultados.:**

Las tendencias de desigualdad fueron divergentes entre estos países. Se observó un grado de avance en cada país, aunque subsistieron grandes brechas entre el quintil de ingresos superiores y el inferior, a pesar de que hubo reformas en la atención de salud. Las brechas en cuanto al índice relativo de desigualdad fueron más elevadas en Colombia (60%), seguido de Paraguay (30%), Perú (20%) y El Salvador (20%).

**Conclusiones.:**

La repercusión que tienen las iniciativas de política nacional sobre la igualdad de acceso a los servicios de salud debería ser objeto de análisis en un futuro. Asimismo, es necesario que se realicen investigaciones en cuanto al seguimiento a nivel nacional y regional de los obstáculos al acceso a los servicios de salud y los factores explicativos que indiquen porqué las personas no acuden a los servicios incluso cuando presentan un problema de salud.

## RESUMO

**Objetivo.:**

Apresentar indicadores das desigualdades socioeconómicas no acesso aos serviços de saúde na Colombia, El Salvador, Paraguai e Peru.

**Métodos.:**

Este estudo transversal utilizou dados de pesquisas domiciliares representativas da realidade nacional na Colómbia, El Salvador, Peru e Paraguai com o objetivo de analisar as desigualdades relacionadas a renda no acesso aos serviços de saúde. Os domicílios que relataram a presença de um problema de saúde (doença/acidente) e nao buscaram cuidados de saúde profissionais foram considerados como domicílios que apresentavam barreiras no acesso aos serviços de saúde. Os indicadores de desigualdade foram o indice absoluto de desigualdade (*slope index of inequality*, SII) e o indice relativo de desigualdade.

**Resultados.:**

As tendencias relativas a desigualdade foram mistas nos quatro países. Todos apresentaram melhorias, mas ainda persistem grandes disparidades entre os quintis com renda mais alta e mais baixa, apesar das reformas nos sistemas de saúde. A desigualdade relativa foi mais elevada na Colómbia (60%), seguida do Paraguai (30%), Peru (20%) e El Salvador (20%).

**Conclusões.:**

O efeito das iniciativas políticas nacionais sobre a equidade no acesso aos serviços de saúde deve ser o tema de análises futuras. Também é preciso pesquisar o monitoramento nacional e regional das barreiras no acesso aos serviços e os fatores que explicam por que as pessoas nao buscam cuidados de saúde, mesmo quando apresentam um problema de saúde.

Achieving equitable access to health services is frequently a goal of health system reform in countries of Latin America (1). In 2014, the Member States of the Pan American Health Organization (PAHO) recognized the need for better access to health services across the Region of the Americas and declared their commitment to strengthening health systems and accelerating progress toward the Universal Access to Health and Universal Health Coverage goals (2). Shortly after, PAHO proposed a Regional monitoring framework that highlights access barriers as key metrics for measuring universal access (3). More recently, in 2019, PAHO introduced the Regional Compact on Primary Health Care for Universal Health (PHC 30-30-30), which explicitly set a goal of reducing barriers to health access by 30% or more by 2030 (4).

This renewed focus on achieving universal access to health underscores the need for effectively measuring equity in access barriers to health services for Regional and national monitoring and evaluation (5). Research on barriers to access health services has been useful in understanding the factors producing inequities in access and informing the design of interventions to reduce those disparities (6 – 9). Recent studies evaluating access barriers in Colombia, El Salvador, Paraguay, and Peru show that 26% – 66% of the population does not seek formal health care when experiencing a health problem (6). The principle reasons for not seeking care were long wait times, availability of resources, e.g., health personnel and medication, and lack of money; greater barriers affected the lower income quintile groups (6, 7). However, the extent to which these countries have improved inequalities in access barriers to health services in recent years is not known. The lack of evidence undermines policy efforts and hinders recommendations and responses by health systems to factors that affect the health needs of individuals, households, and communities (8, 9).

To address these gaps, this study presents summary measures of socioeconomic inequalities in barriers to accessing health services in Colombia, El Salvador, Paraguay, and Peru, countries with distinct health systems, but with a shared goal of improving health access and equity.

## MATERIALS AND METHODS

### Study design and data sources

**FIGURE 1.**
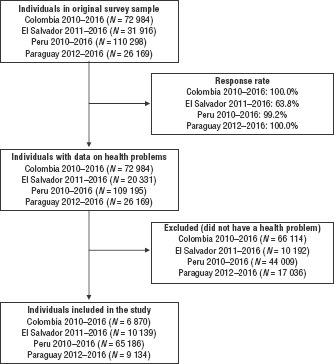
Sample size flow chart for a study of access barriers to health services in four Latin American countries, 2010 - 2016

***Source:*** Authors’ analysis of the National Quality of Life Survey (ECD), 2010 - 2016, Colombia; Multiple Purpose Household Surveys (EHPM), 2011 - 2016, El Salvador; Ongoing Household Survey (EPH), 2012 - 2016, Paraguay; and National Household Survey (ENAHO), 2010 - 2016, Peru.

This was a cross-sectional study based on nationally representative household surveys conducted in Colombia, El Salvador, Paraguay, and Peru in 2010 – 2016 (Figure 1). These countries were selected because they each: (i) have an explicit health system policy goal to provide equitable access to health services; (ii) have taken different approaches to achieving this goal (6, 10); and (iii) have recent, relevant data from population surveys available. Colombia and Peru reforms introduced health financing insurance and economic incentives, while El Salvador and Paraguay used comprehensive primary health care strategies encompassing changes in the governance of health care delivery and health workforce management.

### Variables

The outcome variable was the percentage of individuals reporting barriers to seeking health services, which was measured as previously described (5). Individuals who reported having a health problem (disease/accident) and not seeking professional health care were considered to be facing access barriers. The reasons for not seeking health services were related to: quality of care, delays at the health center, wait time for an appointment, lack of time, shortage of health workers or medications, distance to care, financial limitations, and cultural factors. Household income was a proxy for socioeconomic status. To obtain individual-level estimates, household income was adjusted for household size, as described previously (11).

### Statistical analysis

A review by Wagstaff and colleagues (12) concluded that only two measures meet the minimal requirements for a good inequality measure: the slope index of inequality (SII) and the concentration index (CI). The measures of inequality selected for this study were the SII and relative index of inequality (RII). The SII was selected because its interpretation is more straightforward than the CI (13), and it can be presented in terms of “relative gap,” which can express national and local health inequality targets (14).

The SII represents the regression (*β*) coefficient that shows the relation between the percentage of individuals reporting barriers to accessing health services in each socioeconomic group and the hierarchical ranking of that group according to individual income. Logarithmic data transformation and Maddala’s weighted least-squares regression model were used to account for the non-linearity and intrinsic heteroscedasticity of the data as described previously (15). The SII was interpreted as the absolute change in the percentage of households reporting barriers to access from the highest to lowest hierarchical rank of family income. The ranking is expressed as a value between 0 and 1. That means that the SII can be interpreted as the change in the outcome variable (percentage of individuals reporting access barriers) for a unit change in the independent variable (income level rank), providing an estimation of the absolute gap across the socioeconomic groups from most to least deprived (13). An SII with a negative sign indicates a downward slope with lowest income groups and a higher percentage of individuals reporting access barriers; SII with a positive sign indicates an upward slope with a higher percentage of individuals reporting access barriers in the highest income groups.

The SII has limitations for comparing changes over time because the size of the gap will depend on the scale being used to measure the outcome variable. To address this, the RII was calculated by dividing the SII by the average level of individuals reporting access barriers across all socioeconomic groups. The RII can be interpreted as the proportionate gap relative to that average (13). Values > 1 indicate a concentration of the indicator among the advantaged, and < 1, a concentration among the disadvantaged (13).

The survey sample design was considered when estimating the outcome variable. Expansion factors at the individual level were applied in all cases to calculate national totals. When the unweighted number of observations in a specific subgroup was < 25, results were omitted. For the inequality measures, 95% confidence intervals (95%CI) were used. Stata^®^ Statistical Software: Release 15.1 (StataCorp LP, College Station, Texas, United States) was used for all the statistical analyses. To aid in the interpretation and understanding of these measures, income quintile distributions were visualized graphically through Equiplot charts (International Center for Equity in Health, Federal University of Pelotas, Brazil).

## RESULTS

### Barriers to accessing health services

Table 1 shows a descriptive analysis of the total population by sex, income quintiles, and health insurance coverage. In general, the percentage of individuals in each income group remained stable over time across all countries. Health insurance coverage was higher in Colombia (95.4%), followed by Peru (75.5%), Paraguay (26.4%), and El Salvador (24.1%).

**TABLE 1.** Descriptive analysis of studied population by sex, income quintile level, and health insurance coverage, selected Latin American countries, 2010-2016

Variables	2010	2011	2012	2013	2014	2015	2016
**Males**							
Colombia	48.5	-	48.7	48.7	48.7	48.3	48.6
El Salvador	-	44.5	45.1	44.3	47.5	45.8	47.2
Paraguay	-	-	50.2	49.6	49.4	49.7	50.4
Peru	49.4	49.6	49.3	49.4	49.2	49.2	49.0
**Wealth**							
Colombia							
quintile 1	23.2	-	22.1	22.8	22.8	23.6	23.4
quintile 2	23.0	-	23.5	23.2	23.4	22.8	23.4
quintile 3	21.2	-	20.8	21.0	21.1	20.7	20.5
quintile 4	18.1	-	18.9	18.2	17.8	18.0	18.0
quintile 5	14.5	-	14.8	14.9	14.9	14.9	14.7
El Salvador							
quintile 1	-	22.6	21.4	19.6	18.6	19.4	21.2
quintile 2	-	22.5	21.2	19.4	19.6	19.3	20.1
quintile 3	-	20.7	20.3	19.0	20.5	20.1	19.4
quintile 4	-	18.6	17.9	21.1	20.1	20.7	20.2
quintile 5	-	15.6	19.2	20.9	21.3	20.5	19.1
Paraguay							
quintile 1	-	-	19.4	20.0	19.5	21.5	23.0
quintile 2	-	-	19.6	19.2	20.3	20.2	20.1
quintile 3	-	-	20.4	19.8	20.0	19.7	20.3
quintile 4	-	-	19.8	20.3	20.1	18.9	18.6
quintile 5	-	-	20.9	20.7	20.1	19.7	18.1
Peru							
quintile 1	12.9	12.5	12.4	12.3	12.2	12.3	12.4
quintile 2	19.1	19.1	18.8	18.8	18.7	18.7	18.5
quintile 3	20.9	20.9	20.6	20.9	21.1	21.2	20.8
quintile 4	22.2	22.4	22.6	22.7	23.0	22.7	22.9
quintile 5	24.9	25.2	25.6	25.3	25.0	25.1	25.4
**Health insurance coverage**							
Colombia	88.8	-	90.8	91.4	94.2	94.8	95.4
El Salvador	-	16.9	18.1	19.0	19.9	23.8	24.1
Paraguay	-	-	23.7	23.8	25.6	25.6	26.4
Peru	63.2	64.2	61.8	65.3	68.9	72.8	75.5

***Source:*** Authors’ analysis of National Quality of Life Survey (ECD), 2010 to 2016, Colombia; Multiple Purpose Household Surveys (EHPM), 2011 to 2016, El Salvador; Ongoing Household Survey (EPH), 2012 to 2016, Paraguay; and National Household Survey (ENAHO), 2010 to 2016, Peru.

**FIGURE 2.**
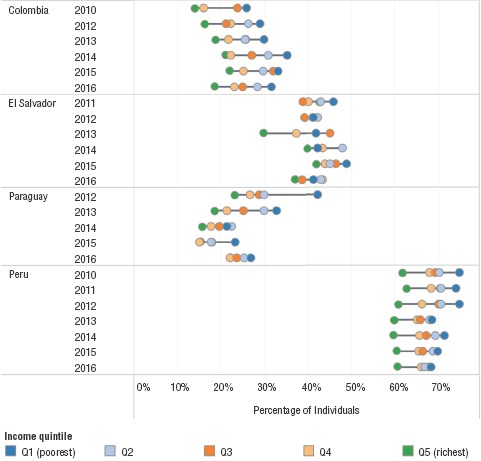
Equiplot of inequality trends of barriers in seeking health services, selected Latin American countries, 2010-2016

*Source:* Prepared by the authors from the study results. The equiplot shows a sequence of dots for each year and country in a line. The farther to the right the sequence of dots is, the higher the percentage of individuals reporting access barriers. Each dot represents one wealth quintile, from the poorest or Q1 (red dot) to the richest or Q5 (blue dot). These two dots are connected by a line; longer lines represent larger absolute inequalities.

Figure 2 shows the percentage of individuals experiencing barriers to accessing health services, by income quintile groups in the four countries. There was a clear socioeconomic gap in the percentage of individuals reporting a health problem but not seeking care, higher among the poorest income quintiles in all four countries.

This socioeconomic gap was particularly pronounced in Colombia and Peru (Figure 2), but diminished in El Salvador, Paraguay, and Peru during the study time period (2010 – 2016). Except for Colombia, the percentage of individuals experiencing access barriers declined slightly by 2016, with the greatest decreases among the lowest income quintiles. By the end of the study period, the percentages of individuals reporting a health care need and experiencing a barrier to care were 25.6% in Colombia, 41.0% in El Salvador, 24.7% in Paraguay, and 65.9% in Peru. Data on the magnitude and type of barriers were previously published in this journal (2).

### Changes in inequalities of barriers to health services

Table 2 shows the RII (relative) and SII (absolute) differences in inequalities between income levels from 2010 to 2016, for the four study countries. In Colombia, there was a clear association between the percentage of individuals reporting access barriers and income levels during all time periods, though RII scores decreased from -0.8 in 2010 to -0.6 in 2016. This socioeconomic inequality was associated with a negative gap between the highest and lowest income groups—61.8% of the national average of the Colombian population reported barriers in 2016 (Table 2). In El Salvador and Peru, most of the RII scores were equal to -0.2, and income-related inequalities remained relatively stable. Socioeconomic inequalities in these countries were also associated with a negative gap of 15.7% and 15.8%, respectively (Table 2). In Paraguay, inequalities decreased substantially, with RII scores declining from -0.7 to -0.3 during the study period.

## DISCUSSION

This study is the first to estimate progress made to reduce socioeconomic inequalities that affect barriers to accessing health services in Latin America. It also shows how countries in the Region can monitor equity in access, as proposed by the PAHO monitoring framework for universal health (3). The findings indicate that, on average, the percentage of individuals who do not seek formal care for a health problem was not reduced substantially during the study period. Also, the percentage experiencing access barriers is high across all countries, particularly in the low-income groups. However, this picture is complex and difficult to summarize due to mixed inequality trends.

In Colombia, absolute inequality remained relatively stable (shown by the SII), while relative inequality decreased over time (shown by the RII). In addition, among the poorest 20%, the percentage of individuals reporting barriers to access increased from 21.4% to 31.9%, and among the richest 20%, from 14.1% to 18.7%.

The opposite was observed in Peru. Absolute inequality decreased and relative inequality was comparatively stable. In the poorest income quintile, the percentage experiencing barriers was reduced from 68.1% to 69.2%; and in the richest, from 62.4% to 61.2%.

**TABLE 2.** Socioeconomic inequalities of barriers in seeking health care, selected Latin American countries

Country	National mean			SII			RII			Relative gap		
	Point value	95% CI		Point value	95% CI		Point value	95% CI		Point value	95% CI	
		Lower	Upper		Lower	Upper		Lower	Upper		Lower	Upper
**Colombia**											
2010	21.4	19.6	23.3	-0.2^a^	-0.2	-0.1	-0.8	-1.2	-0.4	-76.1	-122.2	-37.1
2012	23.1	21.2	25.0	-0.1^a^	-0.2	-0.1	-0.6	-1.0	-0.4	-63.6	-95.1	-36.9
2013	24.7	22.7	26.7	-0.1^a^	-0.2	-0.1	-0.5	-0.8	-0.3	-51.0	-81.0	-25.4
2014	27.8	25.6	30.1	-0.2^a^	-0.2	-0.2	-0.7	-0.8	-0.5	-67.3	-84.7	-52.4
2015	28.8	26.4	31.1	-0.1^a^	-0.2	-0.1	-0.5	-0.7	-0.2	-47.4	-74.2	-24.8
2016	25.6	23.1	28.1	-0.2^a^	-0.2	-0.1	-0.6	-1.0	-0.3	-61.8	-98.7	-31.5
2016-2010	4.2	3.6	4.8	0.0	0.0	0.0	0.1	0.2	0.1	14.3	23.5	5.6
**El Salvador**											
2011	42.5	36.5	48.5	0.0	-0.1	0.0	-0.1	-0.3	0.1	-9.6	-31.3	6.7
2012	41.0	35.3	46.7	0.0	-0.2	0.1	-0.1	-0.4	0.1	-11.2	-44.0	13.6
2013	39.0	33.4	44.7	-0.2^a^	-0.3	-0.1	-0.4	-0.8	-0.1	-43.3	-83.0	-13.6
2014	43.4	38.0	48.8	-0.1	-0.2	0.0	-0.1	-0.5	0.1	-14.9	-45.9	9.2
2015	45.6	39.6	51.5	-0.1	-0.2	0.0	-0.2	-0.4	0.0	-15.6	-42.3	4.8
2016	41.0	35.9	46.2	-0.1^a^	-0.1	0.0	-0.2	-0.3	0.0	-15.7	-32.9	-2.3
2016-2011	-1.5	-0.7	-2.3	0.0	0.0	0.0	-0.1	0	-0.1	-6.0	-1.6	-8.9
**Paraguay**											
2012	32.1	30.7	33.4	-0.2^a^	-0.3	-0.1	-0.7	-1.1	-0.4	-74.2	-110.8	-40.7
2013	27.1	25.7	28.4	-0.2^a^	-0.3	-0.1	-0.7	-1.1	-0.4	-72.1	-109.3	-38.6
2014	20.2	18.8	21.6	-0.1^a^	-0.1	0.0	-0.3	-0.6	-0.1	-33.2	-64.7	-5.9
2015	18.7	17.4	19.9	-0.1^a^	-0.2	0.0	-0.6	-1.2	-0.1	-63.0	-119.4	-13.8
2016	24.7	23.5	25.9	-0.1^a^	-0.2	0.0	-0.3	-0.7	0.0	-29.6	-66.6	4.0
2016-2012	-7.4	-7.3	-7.5	0.2	0.2	0.1	0.4	0.4	0.4	44.6	44.2	44.7
**Peru**											
2010	68.1	67	69.2	-0.2^a^	-0.2	-0.1	-0.2	-0.3	-0.2	-22.6	-29.1	-16.2
2011	68.6	67.5	69.7	-0.1^a^	-0.2	-0.1	-0.2	-0.3	-0.1	-19.8	-25.9	-14.0
2012	67.5	66.4	68.6	-0.2^a^	-0.2	-0.1	-0.3	-0.3	-0.2	-25.8	-32.4	-19.5
2013	65.2	64.1	66.3	-0.1^a^	-0.2	-0.1	-0.2	-0.2	-0.1	-17.7	-23.7	-11.9
2014	66.2	65.1	67.3	-0.2^a^	-0.2	-0.1	-0.2	-0.3	-0.2	-22.9	-29.7	-16.3
2015	65.9	64.8	67	-0.1^a^	-0.2	-0.1	-0.2	-0.3	-0.1	-18.4	-25.2	-11.8
2016	65.9	64.8	67	-0.1^a^	-0.2	-0.1	-0.2	-0.2	-0.1	-15.8	-24.2	-7.6
2016-2010	-2.1	-2.1	-2.1	0.0	0.0	0.1	0.1	0.0	0.1	6.8	4.9	8.7

***Source:*** Prepared by the authors from these results.

a significant at P < 0.01.

95% CI: 95% confidence interval; SII: slope index of inequality; RII: relative index of inequality.

Note: The national means and SII indices are measured in terms of percentage of population who had a health care need, but did not seek care. The inequality gaps are the SII indices as a proportion of the national mean.

In El Salvador, income inequality remained stable in absolute terms, but relative inequality increased during the study period. Both the richest and poorest income quintiles saw decreases, from 42.5% to 41.0% and from 43.2% to 37.4%, respectively.

By contrast, Paraguay saw substantial decreases in socioeconomic inequalities, both absolute and relative. The reduction was most rapid among the poorest 20%, from 42.8% to 27.1%; less so among the richest 20%, from 23.5% to 22.4%.

All four countries showed improvement, but large inequality gaps persisted between the highest and lowest income population. Although, the RII and the SII are good measures of relative and absolute inequality (12, 13), they can lead to differing conclusions about the direction of change over time, depending on the trajectory of the indicator overall (13). The findings of this study demonstrate this issue. For example, in Peru, the percentage of individuals reporting access barriers among the poorest 20% decreased from 75.6% to 69.2%, and among the richest 20%, from 62.4% to 61.2% (Figure 2). As a result, absolute inequality between the two was reduced substantially, from 13.2 to 8.0 percentage points; but the relative inequality actually increased slightly, from 82.5 to 88.4. Such trends mirror those observed with the SII and RII.

To improve access to health services, especially among the poorest, Colombia and Peru expanded insurance coverage (6). Colombia introduced a social security system based on the managed competition model. Peru passed the Universal Health Insurance Law to expand coverage of health insurance (6). The results echo those of other studies that found limited improvements despite expanded insurance, with individuals in low-income groups still facing substantially more barriers than the richest (1, 16). These failings support the view that segmenting health insurance schemes across diverse population groups based on socioeconomic status leads to inequalities (17, 18).

El Salvador and Paraguay sought to improve access through a comprehensive primary health care strategy that gave priority to the public sector and directed funding toward vulnerable social groups. Since 2009, El Salvador has been implementing comprehensive reforms based on primary health care to guarantee universal and equitable access to health care (6). In 2008 – 2013, Paraguay made substantial investments in primary health care, including the creation of first level of care units, consolidation of community primary care teams, and elimination of direct payments at the point of service (6). For these two countries, insurance coverage has not increased much, yet they have substantially reduced out-of-pocket expenditures on first-level health services and fewer people attribute lack of access to a financial barrier (6). Our study findings show that improvements made in Paraguay toward equality mirror evidence suggesting that in countries where primary health care is a high priority, the odds of achieving equitable health care are better (19).

A literature search for previous studies on socioeconomic inequalities and barriers to accessing health services did not return any for Colombia, El Salvador, Paraguay, or Peru. However, it did find a few comprehensive studies reporting trends similar to those observed in this study. A longitudinal study in Colombia from 2003 – 2008 compared indicators of health inequality, and found that both concentration indices and horizontal measures of inequality improved with health insurance affiliation, access to medicine, and curative services (20). However, striking gaps were revealed in the proportion of health service utilization among the income quintile groups, with significantly fewer preventive and curative, as well as outpatient and inpatient health services used by the poorest. A cross-sectional study of factors influencing access in two municipalities of Colombia found that geographic and financial factors and obtaining required insurance authorization were the greatest barriers to accessing health services. The segmented nature of the Colombian health system and the role of insurance companies appeared to be related to these results (21).

In Peru, a national health accounts study showed that although self-reported health problems increased from 51.3% in 2004 to 61.5% in 2012, utilization of health services increased much less, from 31.0% to 32.7% (22). In addition, the percentage of those who reported seeking care for a health problem at a pharmacy (self-medication) increased from 26.3% to 29.3%. Similarly, demand for formal care fell among beneficiaries of SIS (Seguro Integral de Salud) and EsSalud, indicating a decline in the system’s effectiveness and ability to meet the population’s health needs (23). Another inequality study in Peru looked at concentration indices in 2012 and reported that distribution of health services utilization suggests a benefit for the richest populations, except at the Ministry of Health’s non-hospital facilities (24).

The strengths of this study are its use of household surveys with a nationally representative sample size and inequality measures based on the entire socioeconomic gradient across all populations. The study looked at inequality in absolute and relative terms because both can move in opposite directions when the mean is changing over time.

### Limitations.

The study’s main limitations involved analysis of explanatory factors, specifically reasons for not seeking formal health care when experiencing a health problem. Current national household surveys include explanations such as: “did not have time,” “did not have money to pay for services,” and “considered the problem unimportant,” but do not give the respondent the opportunity to explain the circumstances behind the reason for not seeking care. However, these responses are indicative of and useful for monitoring trends in barriers to accessing care (9, 21). Also, survey data should be complemented with qualitative studies. Mix methods approaches can improve the understanding of the underlaying factors that influence access to health services and provide useful insights for policymaking decisions (25 – 27).

Given the differences in the surveys across Latin American countries, comparability among countries becomes challenging. While this study did not aim to compare inequalities in access to health services between countries, careful revision and selection of variables across surveys was conducted, as described previously (6). Another limitation was the use of cross-sectional data, which restricts the ability to infer causality. Health system reforms to improve equity in access may take long periods to effect change, and time required to accurately evaluate such effects will vary. Furthermore, improvements in socioeconomic factors outside the health sector contribute to positive changes in health equity and access, and therefore, should be considered as well. Nevertheless, the methodology used in this study does not aim at determining causality. Rather, the study seeks to describe and explain trends in income-related inequalities in barriers to access health services over time.

Finally, the study’s measure of access was based on barriers between the population and an initial contact with health services. We did not capture access in its broad domain, i.e., from realizing health services were necessary through the use of services, including treatment and follow-up, satisfaction with care, quality of services, and health outcomes (28). Therefore, it is possible that certain unevaluated aspects of access did not follow the same inequality trends observed. That said, similar studies found similar trends.

### Recommendations

There are many factors that influence an individual’s ability to access health services, and these in turn, affect inequality trends. Evaluation of specific factors was beyond the scope of this study, but warrants future research. The effects of health-sector reform strategies and policy initiatives on the equity of access to health services should be evaluated and barriers to access need to be monitored. There are few comprehensive studies that inform policymakers on effective ways to offer universal access to health care (8). Most research focuses on specific components of the health system, such as the effect of financing or service delivery mechanisms on patterns of health care utilization as a proxy of access (e.g., visit rates) (8). However, several authors have noted important limitations to this approach (28 – 30): (i) those who use health services may have overcome substantial barriers; (ii) measuring health services use alone may mask significant obstacles faced by individuals who need care, but fail to use it.; and (iii), lack of use could be an informed choice or personal preference, and does not necessarily mean poor access. Health care access is a complex and multidimensional concept comprised of different, distinct dimensions that need to be considered, e.g., availability of health resources, location of health care centers, convenient office hours, gender, religion, etc.

Consequently, recommendations and policy responses give little attention to other factors influencing how and whether health systems respond to the health needs of individuals, households, and communities (8). Analytical frameworks that acknowledge the multidimensionality of access are increasingly being presented to explain the causes of inequities in access to health care, and have proven useful to informing interventions that reduce disparities (8).

## CONCLUSIONS

The link between wealth and access to health services is well documented internationally. The findings of this study reinforce the association. Across the four study countries— Colombia, El Salvador, Paraguay, Peru—the percentage of the population faced with barriers to access was persistently high, particularly among low-income individuals and in spite of targeted health sector reforms. The mixed progress in inequality trends reflects the complexity and multidimensionality of access to health care. Countries that seek to achieve more equitable access require interventions that address modifiable determinants of access pertaining to the health system, individuals, and communities. The measures explored by this study can help develop the evidence base for reducing inequalities by monitoring equity at the local, national, and international levels.

### Author contributions.

NH and EB designed the study. NH carried out the calculations and took the lead in writing the manuscript, in consultation with EB and ADR. Overall direction and planning were overseen by ADR. All authors provided critical feedback and helped shape the research, analysis, and manuscript. All authors reviewed and approved the final version.

### Acknowledgments.

The authors would like to thank Ricardo Sánchez for his contributions to this article.

#### Disclaimer.

Authors hold sole responsibility for the views expressed in the manuscript, which may not necessarily reflect the opinion or policy of the *RPSP/PAJPH* and/or PAHO.
